# Development and Validation of a Multiplex Flow Cytometry‐Based PBMC Assay for Immunomodulator Screening

**DOI:** 10.1155/jimr/5535208

**Published:** 2026-08-04

**Authors:** Regine Brox, Holger Hackstein

**Affiliations:** ^1^ Department of Transfusion Medicine and Hemostaseology, University Hospital Erlangen, Erlangen, Germany, fau.eu

## Abstract

The identification of novel compounds capable of stimulating or suppressing immune responses is critical for advancing immunotherapy and improving our understanding of immune regulation. However, traditional screening approaches are often limited by low throughput, poor physiological relevance, and insufficient resolution to detect subtle immunomodulatory effects. In this study, we introduce a validated and robust multiplexed flow cytometry‐based functional screening assay that enables efficient and precise identification of immunomodulatory compounds, while addressing the key limitations of conventional methods. By using human peripheral blood mononuclear cells (PBMCs) and defined stimulation conditions, the assay mimics complex immune interactions in a physiologically relevant context. It simultaneously monitors T‐ and B‐cell responses across multiple functional readouts, including T‐cell proliferation (carboxyfluorescein succinimidyl ester [CFSE]), activation (CD25, CD71), and B‐cell costimulatory marker expression (CD86), enabling an in‐depth assessment of immune modulation within heterogenous cell populations. The incorporation of robust statistical validation using the Z‐factor further supports assay reliability and reproducibility. In a pilot screen with 295 immunology‐related compounds, the assay demonstrated its ability to identify known immunomodulators with distinct functional profiles. This multiplex functional screening approach bridges the gap between mechanistic immunology and drug discovery by enabling systematic identification and characterization of immunotherapeutic candidates, while providing new insights into the complex dynamics of immune regulation.

## 1. Introduction

The immune system is essential for maintaining body homeostasis and regulating cellular functions by safeguarding the body against infections and diseases. T and B cells are central to the adaptive immune response, each employing highly specialized receptor‐mediated signaling pathways that orchestrate their activation, proliferation, and differentiation [1]. Dysregulation of these finely tuned processes can lead to autoimmune diseases [2], malignancies [3], and chronic inflammation [4], highlighting them as crucial targets for therapeutic intervention. Several classes of drugs, such as glucocorticoids, small‐molecule inhibitors, cytokines, immunoadjuvants, and biological agents, are available for the treatment of such diseases [5, 6]. However, current therapies often grapple with challenges such as resistance, limited specificity, and adverse side effects, underscoring the need for compounds that can more precisely modulate immune responses—either by boosting or suppressing specific pathways.

Despite the importance of identifying such compounds, current immunological methods often depend on limited endpoints that do not fully capture the dynamic interactions between immune cells. Additionally, they lack standardization based on pharmacological criteria, which is essential for ensuring the reliability and reproducibility of the experimental results. This study describes the development and validation of a cell‐based flow cytometry screening assay for profiling the immunological effects of small molecules across different stages of T‐ and B‐cell signaling. Flow cytometry offers a unique combination of sensitivity, specificity, and versatility, making it an ideal platform for functional screening of immunomodulatory compounds [7]. By enabling simultaneous multiparametric analysis of thousands of cells, this technology allows us to assess subtle changes in cell surface markers, intracellular signaling events, and other functional parameters in real time [8, 9]. However, applying flow cytometry to immunomodulator screening requires careful assay optimization and validation to ensure reproducibility and reliable data acquisition. We therefore combined flow cytometry with specific, easily assayable markers for T‐ and B‐cell activation, together with consistent gating strategies and sample preparation workflows. Additionally, we incorporate the Z‐factor as a rigorous pharmacological criterion, ensuring the precise detection of subtle immunological shifts induced by potential modulators. Moreover, the utilization of human peripheral blood mononuclear cells (PBMCs) from both male and female donors provides a physiologically relevant primary cell model that preserves complex immune cell interactions more effectively than immortalized cell lines. This gender‐inclusive approach not only accounts for interindividual variability but also enhances the translational relevance of our findings and the comprehensive understanding of drug efficacy by reflecting the full spectrum of immune responses [10]. Ultimately, the pilot screening of the commercially available DiscoveryProbe Immunology/Inflammation Compound Library, which consists of 295 compounds related to immunology and inflammation, demonstrated that the multiplex flow cytometry‐based functional screening assay marks a significant advancement in immunomodulatory research and provides a reproducible and versatile approach for identifying potential immunomodulatory compounds.

## 2. Materials and Methods

### 2.1. Blood Samples

Sampling of residual blood samples from healthy adult blood donors is conducted after participants give written consent during a regular blood donation within the Department of Transfusion Medicine and Hemostaseology of the University Hospital Erlangen. Blood donors are routinely screened according to standard blood donation eligibility criteria and are therefore considered healthy individuals without known acute infections or significant underlying diseases at the time of donation. Throughout the study, PBMCs were obtained from 11 independent healthy blood donors aged 27–43 years (66.7% male, 33.3% female; median age 33 years). Each biological replicate represents an independent experiment performed with PBMCs derived from a different donor. The local ethics committee has approved the conduct (# 346_18B and # 343_18B).

### 2.2. T‐Cell Activation

PBMCs were enriched from leukocyte reduction system (LRS) chambers derived from routine blood donations by density gradient centrifugation. For T‐cell activation, a 96‐well flat‐bottomed plate was coated with 0.5 μg/mL anti‐CD3 (HIT3α; BioLegend) or phosphate‐buffered saline (PBS) (unstimulated cells) for 2 h at 37°C. Cells were washed with PBS, and 180 μL of carboxyfluorescein succinimidyl ester (CFSE)‐labeled PBMCs (1.6 × 10^6^ cells/mL) were seeded on the plate together with 20 µL either vehicle (PBS/0.5%DMSO) or test compound and cultivated in complete cell culture medium consisting of IMDM (Gibco) supplemented with 10% AB serum (Capricorn), 1% L‐Glutamin (Gibco), 1% penicillin/streptomycin (Gibco), and 55 µM 2‐Mercaptoethanol (Roth). T‐cell activation was acquired 72 h later via flow cytometry and analyzed using FlowJo software.

### 2.3. Flow Cytometry and Antibodies

Cells were stained with CFSE using the CFSE Cell Division Tracker Kit (BioLegend) in accordance with the manufacturer’s protocol. Briefly, PBMCs were resuspended with CFSE in PBS (final concentration, 1 μM) at 1 × 10^7^ cells/mL and incubated for 5 min at 37°C in the dark. Staining was quenched by washing cells with five times the staining volume of PBS with 10% fetal calf serum (FCS).

Extracellular antigens were stained with antibodies for 30 min at 4°C in PBS containing 2% FCS. The following antibodies from BioLegend were used: anti‐CD3‐Alexa Fluor 700 (OKT3), anti‐CD19‐APC/Fire 750 (SJ25C1), anti‐CD25‐APC (BC96), anti‐CD71‐PE (CY1G4), and anti‐CD86‐Brilliant Violet 605 (IT2.2). SYTOX Blue dead cell stain (Thermo Fisher) was added at a final concentration of 50 nM 5 min before analyzing the samples via flow cytometry. The live/dead gate was set using an unstained control and an ethanol‐killed PBMC control (Sytox Blue–positive) to define dead cells; Sytox‐negative events were subsequently gated as live cells. Samples were evaluated with a flow cytometer (CytoFLEX S, Beckman Coulter), and data were analyzed with computer software (FlowJo 10.10.0, TreeStar). CFSE is an effective cell permeant fluorescent dye to monitor lymphocyte division [11]. CD71, also known as the transferrin receptor, is upregulated early in T‐cell activation and is associated with increased metabolic activity and iron uptake, which are critical for cell growth [12]. CD25, the IL‐2 receptor α‐chain, is a key cell surface marker that supports sustained proliferation and differentiation through IL‐2 signaling [13] Although CD25 is also expressed by regulatory T cells, in this assay, it primarily reflects IL‐2–dependent activation of T cells within the mixed PBMC population. CD86 is a costimulatory molecule that interacts with CD28 on T cells, providing essential signals for T‐cell activation and survival [14].

### 2.4. Z‐Factor Measurement

For assay quality assessment, the Z‐factor of our functional screening assay was calculated on three different days according to the method described. The Z‐factor is a dimensionless screening window coefficient that was defined by Zhang et al. [15] as a statistical tool for the quantitative evaluation of the assay quality. The quality is considered excellent if the Z‐factor is greater than 0.5. Briefly, 180 µL CFSE‐labeled PBMCs and 20 µL vehicle were seeded into a 96‐well plate and incubated with negative controls (unstimulated cells) and positive controls (anti‐CD3 stimulated cells) in an interleaved plate setup for 72 h. The median fluorescent intensity (MFI) of the activation markers as described above was recorded, and the Z‐factor was calculated according to the following formula

Z=1−33σN+σPμN−μP,

where *σ* denotes the standard deviation (SD) and *µ* the mean value. N and P indicate the negative and positive controls, respectively.

### 2.5. Determination of IC_50_ Values for Known Immunosuppressive Drugs

For the determination of the half maximal inhibitory concentration (IC_50_) of the known immunosuppressive drugs Dexamethasone, Cyclosporin A, Rapamycin, Tacrolimus, and Mycophenolate mofetil (Biozol), T‐cell activation was performed as describe above, and cells were incubated with increasing concentrations of test compound (ranging from 10 pM to 10 µM) or vehicle. Representative dose–response curves for each drug were plotted based on the percentage of unstimulated (0%) and stimulated (100%) cells. These curves were used to calculate the IC_50_ values using nonlinear regression analysis.

### 2.6. Pilot Screen With Immunology/Inflammation Compound Library

**Figure 1:**
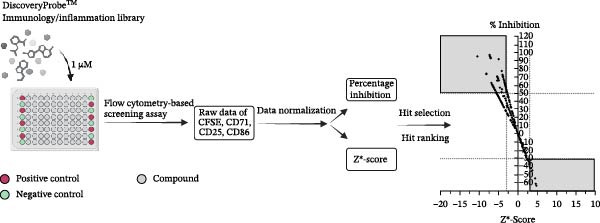
Workflow of the pilot screen with immunology/inflammation compound library using the functional flow cytometry‐based screening assay for immunomodulator identification. Created in BioRender. Heger, L. (2025) https://BioRender.com/xx9wo4j.

The commercially available DiscoveryProbe Immunology/Inflammation Compound Library (APExBIO) consisting of 295 Immunology/Inflammation‐related compounds was used for the pilot screen (Figure 1). The library compounds were provided in a DMSO solution and added at a final concentration of 1 µM. The final concentration of DMSO was 0.5%. The T‐cell activation was measured after 72 h, as described above. For reproducibility and data normalization, negative and positive controls were positioned alternately in the outer columns of the 96‐well plate. The compound library screen comprised two independent screening runs, each performed using PBMCs from a different donor. For each activation marker (CFSE, CD71, CD25, CD86), raw data from both runs were processed by calculating the mean of percentage inhibition and the robust Z‐score (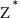
‐score) for normalization. Percentage inhibition was calculated relative to the positive (activated) and negative (nonactivated) controls, allowing for a biologically interpretable measure of compound effect. In parallel, the 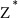
‐score was calculated using the median and median absolute deviation (MAD) of the positive controls. Using both approaches in tandem is essential for reliable hit identification. While percentage inhibition provides a clear biological frame of reference, the 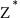
‐score highlights statistically significant outliers within each plate, independent of absolute scale or interplate variation.

Percent inhibition = Value sample−Median of negative controlMedian of positive control−Median of negative control×100,

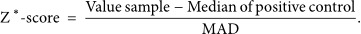




MAD = 1.4826  ^∗^ Median (|Value positive control – Median of all positive controls|).

Compounds were defined as hits if they met both of the following criteria: percentage inhibition ≥ +50% (inhibitors) or ≤ –30% (activators), and a robust 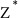
‐score < –3 or > +3.

## 3. Results

### 3.1. Development of a Functional Flow Cytometry‐Based Screening Assay

**Figure 2:**
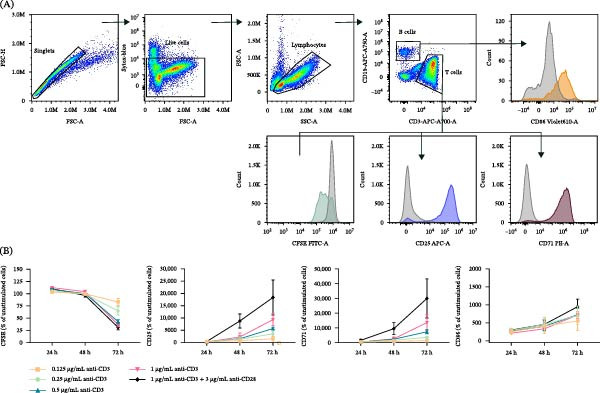
(A) Representative gating strategy of T‐cell screening assay. Human PBMCs were first gated to exclude doublets (FSC‐H vs. FSC‐A), followed by gating on live cells using a viability dye. Lymphocytes were gated based on their characteristic scatter patterns. Within the lymphocyte gate, T cells (CD3^+^CD19^−^) and B cells (CD19^+^CD3^-^) were distinguished. T‐cell activation and proliferation were assessed by median fluorescent intensity (MFI) analysis of CD71, CD25, and CFSE, while B‐cell activation was evaluated by CD86 expression. (B) Assay optimization. PBMCs were stimulated with different concentrations of anti‐CD3/anti‐CD28 and proliferation and activation were measured at different time points (24, 48, 72 h). Orange: 0.125 µg/mL anti‐CD3; green: 0.25 µg/mL anti‐CD3; blue: 0.5 µg/mL anti‐CD3; pink: 1 µg/mL anti‐CD3; black: 1 µg/mL anti‐CD3 + 3 µg/mL anti‐CD28. Data are expressed as percentage of unstimulated PBMCs (set to 100%) and are presented as median ± SD of three independent experiments (*n* = 3), each performed with 12 technical replicates.

The discovery and development of novel immunomodulators are pivotal in pushing the boundaries of medical treatment, allowing for more targeted, effective, and safer interventions in the management of a wide array of diseases. Currently available immunological methods are limited in resolution, multiplexing, and sensitivity, making them suboptimal for immunomodulator screening. To address these unmet needs, we have established a multiplex flow cytometry‐based screening assay that enables quantitative evaluation of compound‐induced effects on T‐ and B‐cell immune function. The assay consists of two main components: (1) human PBMCs as a physiologically relevant *in vitro* culture system, stimulated with anti‐CD3 antibodies to mimic T‐cell receptor (TCR) activation, and (2) specific and easily assayable markers for T‐cell proliferation (CFSE) and activation (CD25 and CD71) and B‐cell activation via T‐cell–dependent mechanisms (CD86), thereby capturing complementary aspects of immune activation and increasing confidence in hit identification. Assessing CD86 expression on B cells additionally provides insights into costimulatory interactions and antigen presentation dynamics within the mixed PBMC culture system. The marker panel was intentionally kept compact to maintain assay robustness and reproducibility for compound screening while still capturing key functional aspects of immune activation. Although high‐dimensional flow cytometry panels can provide deeper immunophenotypic characterization, a limited set of well‐established functional markers was chosen to minimize experimental complexity and ensure reproducibility across screening experiments. The gating strategy used to identify and analyze these populations is depicted in Figure 2A. In addition, Sytox Blue dead cell stain was measured as a built‐in orthogonal cell viability readout that helps to triage false positives due to compound toxicity. The combined use of multiple functional markers improved reliability of hit identification and facilitated downstream validation. While anti‐CD3/CD28 costimulation provides a more physiologically complete activation signal for T cells, anti‐CD3 stimulation alone enables the detection of subtle immune‐modulating effects while avoiding the confounding influences of strong costimulatory signals [16]. This strategic decision aligns with the assay’s objectives and makes it particularly well‐suited for drug discovery applications that require identifying both immune‐enhancing and immune‐suppressive compounds. We performed initial experiments to define the sufficient concentration of T‐cell stimulus and the appropriate time point for simultaneous measurement of all activation markers (Figure 2B). Incubation of PBMCs with 0.5 µg/mL anti‐CD3 for 72 h yields a robust signal window suitable for detecting both immunostimulatory and immunosuppressive effects.

### 3.2. Validation of the Robustness of the Assay Signal Window

**Figure 3:**
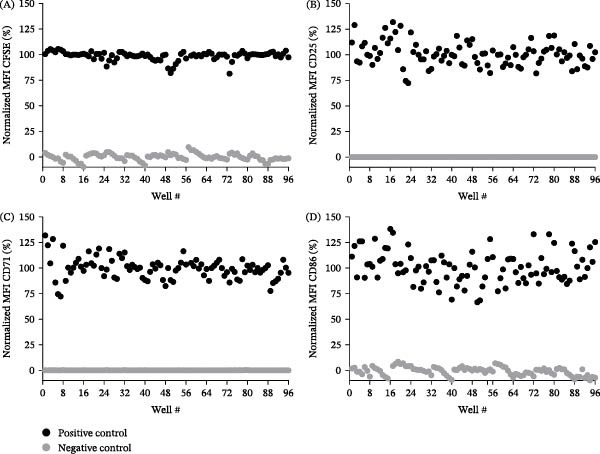
Assay signal distribution of CFSE (A), CD25 (B), CD71 (C) on T cells, and CD86 (D) on B cells following activation with anti‐CD3 for 72 h. Each plot shows the scatter distribution of normalized mean values for negative (gray) and positive (black) controls from three independent experiments performed on separate days. Positive and negative controls were arranged in alternating rows across the 96‐well plate, and their positions were shifted between experiments to minimize positional bias. Corresponding Z‐factor values calculated from these control distributions are indicated in the Results section.

We employed a procedure to evaluate the robustness and reliability of the assay by capturing data from positive and negative controls in an interleaved 96‐well plate setup in 3 independent experiments conducted on separate days. The Z‐factor (Z) was calculated for CFSE, CD71, CD25, and CD86 as a statistical measure of the assay signal window. The Z‐factors of our parameters were analyzed to be 0.77, 0.68, 0.65, and 0.41, respectively, predicting a good performance in high‐throughput screenings (HTSs) (Figure 3). Raw MFI values are provided in Supplementary Table S1.

### 3.3. Determination of IC_50_ Values for Known Immunosuppressive Drugs

To further validate the pharmacological relevance of the screening assay, we tested a panel of well‐established immunomodulatory compounds, including dexamethasone, cyclosporin A, rapamycin, and mycophenolate mofetil. These known immunosuppressive drugs were selected not only for their clinical relevance but also for their distinct mode of action within the T‐cell signaling cascade, enabling a comprehensive evaluation of the assay’s sensitivity to diverse mechanisms of immunosuppression (Figure 4A). Dose‐response curves to assess the half‐maximal inhibitory concentrations (IC_50_) and maximal observed inhibition (Emax, % relative to stimulated control) for all functional readouts are depicted in Figure 4B–F. Dose‐response analysis revealed compound‐specific inhibition profiles for each immune marker with distinct potencies and efficacies. These results highlight the assay’s ability to resolve immunomodulatory effects mediated through diverse targets within the T‐cell signaling cascade and support its suitability for compound library screening to identify novel modulators of immune cell function.

Figure 4(A) Simplified schematic of the T cell signaling cascade and sites of action of known immunosuppressive drugs. Created in BioRender. Heger, L. (2025) https://BioRender.com/ssmk8hk. Dose response curves of dexamethasone (B), cyclosporin A (C), rapamycin (D), and mycophenolate mofetil (E) on T‐ and B‐cell activation markers. Data represent compound‐induced inhibition of T cell proliferation (CFSE), T‐cell activation (CD71, CD25), and B‐cell activation (CD86), normalized to unstimulated (0%) and anti‐CD3 stimulated (100%) cells. Experiments were performed in three independent experiments (*n* = 3), each in duplicate. (F) pIC_50_ values and maximal inhibitory effects (Emax) of immunosuppressive drugs. pIC_50_ values were calculated from dose–response curves using a four‐parameter logistic (variable slope) fit. Emax represents the maximal observed inhibition (% relative to stimulated control) for each marker. Data are presented as mean ± standard deviation (SD).

**Figure:**
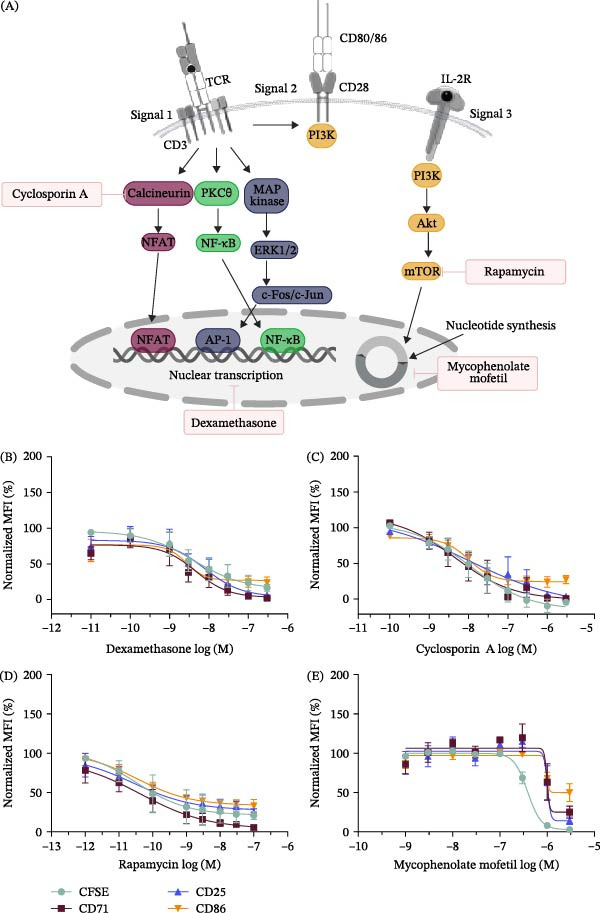

**Figure:**
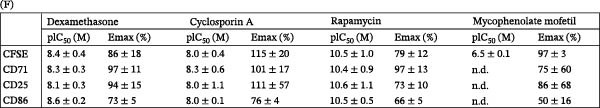

### 3.4. Pilot Screen With Immunology/Inflammation Compound Library

**Figure 5:**
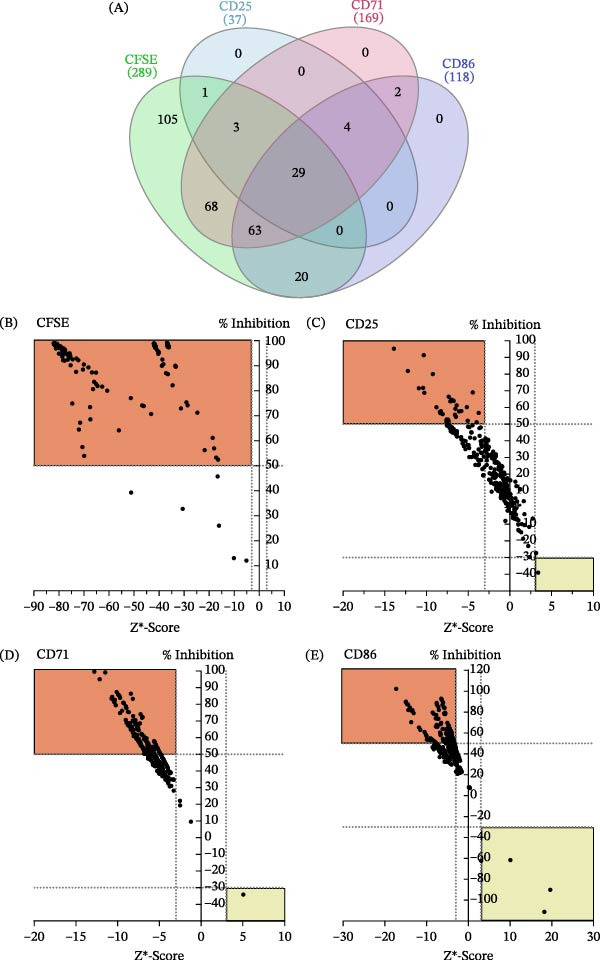
Identification and overlap of T‐cell modulatory compounds from pilot screen across multiple activation markers. (A) Venn diagram illustrating the overlap of identified hits across the four markers. Scatter plots (B: CFSE, C: CD25, D: CD71, and E: CD86) showing percentage inhibition versus 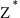
‐score for each activation marker. The dotted lines represent the cutoff points for hit selection. Compounds were defined as potential hits if they showed a percentage inhibition of ≥ +50% (inhibitors) or ≤ −30% (activators) and had a 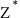
‐score < −3 or > +3 (gray boxes). Inhibitory effects are highlighted in orange, whereas activating effects are shown in yellow for the respective marker readouts. Each compound was tested at a single concentration in two independent screening runs, and the median value of both experiments was used for hit identification.

Next, the established functional flow cytometry‐based assay was used to perform a pilot screen using the immunology‐focused DiscoveryProbe Immunology/Inflammation Compound Library consisting of 295 small molecules with known or predicted immunomodulatory activity. The library comprises bioactive compounds targeting diverse pathways involved in immune regulation and inflammatory signaling, and the overall screening workflow is summarized in Figure 1. To improve the reliability of hit identification and overcome the limitations of single‐step HTS, a two‐step duplicate screening approach was implemented. Each compound was tested in two independent assay runs, and a compound was only classified as a positive hit if it demonstrated activity in both replicates. This strategy significantly enhanced the discrimination between true hits and false positives. For all measurements, the median value of the two experiments was used to ensure robustness against variability and potential outliers. Raw screening data were analyzed using percentage inhibition and robust Z‐score (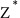
‐score), a statistically robust variant of the classical Z‐score. Compounds were defined as potential hits if they showed a percentage inhibition of ≥ +50% (inhibitors) or ≤ −30% (activators) and had a 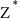
‐score < −3 or > +3 (Figure 5B–D). Activity thresholds were empirically established based on the response range of known immunomodulatory compounds within the assay, while 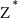
‐score thresholds were selected according to commonly used screening criteria for identifying statistically significant outliers. This dual strategy increases confidence in true hits by combining biological relevance with statistical rigor. Out of 295 compounds, 289 hits were obtained for CFSE (98%), 37 for CD25 (13%), 169 for CD71 (57%), and 118 for CD86 (40%). The high proportion of CFSE hits reflects the high sensitivity of proliferation‐based readouts to perturbations in T‐cell activation and cell cycle progression. As CFSE staining is performed at the beginning of the assay and the dye passively diffuses into the cells, this readout is less susceptible to technical variability compared to antibody‐based markers introduced during the final staining procedure. In this screening context, CFSE therefore functions as a sensitive initial readout, while additional activation markers and viability staining help distinguish general cytostatic effects from more specific immunomodulatory activities. To visualize the overlap of hits between markers, a Venn diagram was generated (Figure 5A) [17]. Of the total number of identified hits, 10% of compounds were active across all four markers (CFSE, CD71, CD25, and CD86), indicating broad and consistent immunomodulatory activity. An additional 24% of compounds showed activity in three markers, while 31% were positive in two markers. The remaining 35% of hits were unique to a single marker, reflecting potential specificity for distinct activation pathways. This distribution highlights both shared and marker‐specific compound effects, underscoring the added value of a multiplexed marker approach in uncovering diverse immunomodulatory profiles.

The pilot screen successfully identified multiple compounds with known immunosuppressive activity as hits, confirming the assay’s robustness and pharmacological relevance (Table 1). Notably, several well‐established immunomodulators – such as cyclosporine A, dexamethasone, rapamycin, everolimus, mycophenolate mofetil, tacrolimus, and pimecrolimus‐ were among the top‐ranked compounds, defined as those exhibiting inhibitory activity on three or more activation markers. In addition, a considerable number of glucocorticoids and cyclooxygenase (COX) inhibitors were identified, which are known to suppress T‐cell responses by directly inhibiting T‐cell activation, proliferation, and survival (glucocorticoids), or indirectly by reducing proinflammatory prostaglandin signaling that supports T‐cell activation (COX inhibitors). This further supports the assay’s ability to detect compounds with diverse anti‐inflammatory and immunosuppressive mechanisms of action. These findings validate the assay as a reliable platform for the discovery and profiling of both known and novel immunomodulatory compounds.

**Table 1:** Summary of top‐ranked immunomodulatory compounds identified in the pilot screen, including their ‐score and percentage inhibition of immune activation markers (CFSE, CD25, CD71, CD86).

Chemical name	Pathway	Information	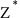 ‐score/percentage inhibition (%)			
			CFSE	CD25	CD71	CD86
Cyclosporine	i.a. Calcineurin–NFAT	Immunosuppressant drug	−16.4/52	−4.0/51	−8.1/61	−5.4/64
SC 144	JAK/STAT	Gp130 inhibitor	−21.8/56	−5.2/60	−9.5/76	−4.9/50
Eltrombopag olamine	JAK/STAT	C‐mpl agonist	−69.9/54	−6.6/56	−7.3/69	−7.8/66
Cilostazol	Metabolism	PDE3 inhibitor	−72.0/64	−6.5/54	−7.4/68	−7.5/66
GS‐9620	Microbiology and virology	TLR‐7 agonist	−73.1/88	−6.5/59	−6.8/59	18.2/−111
Flumethasone	GPCR/G protein	Glucocorticoid receptor agonist	−64.6/82	−7.3/66	−9.7/84	−14.7/88
Triamcinolone	GPCR/G protein	Synthetic glucocorticoid agonist	−74.5/92	−5.1/52	−8.9/78	−13.4/82
Halcinonide	Immunology/inflammation	Anti‐inflammatory agent	−70.4/88	−6.4/61	−9.3/81	−13.6/81
Resiquimod	Immunology/inflammation	Immune response modifier	−67.6/73	−9.2/80	−8.8/76	19.6/‐90
Halobetasol propionate	Metabolism	Phospholipase (e.g., PLA) inhibitor	−60.7/80	−7.0/64	−9.8/85	−15.0/90
Budesonide	GPCR/G protein	Anti‐inflammatory corticosteroid	−66.0/84	−6.7/64	−9.9/86	−14.4/87
Beclomethasone dipropionate	GPCR/G protein	Corticosteroid	−66.4/81	−6.1/58	−10.1/87	−14.4/82
Difluprednate	Glucocorticoid pathway	Corticosteroid	−62.7/82	−5.8/58	−9.2/81	−14.0/84
Clobetasol propionate	GPCR/G protein	Glucocorticoid receptor agonist	−65.1/83	−7.0/65	−9.9/86	−15.0/90
Dexamethasone acetate	GPCR/G protein	IL receptor modulator	−37.2/87	−8.3/58	−10.7/83	−6.1/89
Prednisolone acetate	GPCR/G protein	Synthetic corticosteroid drug	−38.3/91	−8.8/60	−10.2/79	−5.6/79
Fostamatinib	Tyrosine kinase	Spleen tyrosine kinase (Syk) inhibitor	−28.6/74	−10.3/69	−10.6/85	−6.2/87
Sodium salicylate	Immunology/inflammation	NF‐κB inhibitor	−41.5/98	−8.4/57	−8.7/68	−4.7/66
Pirfenidone	TGF‐β/Smad signaling	TGF‐β production inhibitor	−18.1/57	−10.9/71	−10.3/83	−5.8/79
INF39	Immunology/inflammation	Irreversible NLRP3 inhibitor	−42.0/99	−7.3/52	−8.8/68	−3.4/51
CORM‐3	CO signaling	Exhibits anti‐inflammatory/cardioprotective effects	−38.7/92	−7.6/51	−8.7/69	−5.6/78
(S)‐Ketoprofen	Neuroscience	COX‐1 and COX‐2 inhibitor	−81.9/99	−1.3/26	−5.8/52	−6.7/45
Fenoprofen calcium hydrate	Immunology/inflammation	Nonsteroidal, anti‐inflammatory antiarthritic agent	−41.5/97	−7.5/50	−8.4/66	−4.7/67
Tempol	Immunology/inflammation	Superoxide scavenger	−41.8/98	−7.5/51	−7.7/60	−3.8/54
Mepyramine maleate	Neuroscience	Inverse agonist for the H1 receptor	−41.8/98	−7.4/52	−8.2/63	−3.4/51
Alprostadil	GPCR/G protein	Vasodilator	−36.5/87	−10.4/72	−10.4/81	−4.7/66
Mometasone furoate	GPCR/G protein	Glucocorticoid receptor agonist	−34.6/82	−8.4/57	−10.6/83	−5.8/84
Fluticasone propionate	GPCR/G protein	High affinity, selective glucocorticoid receptor agonist	−24.7/71	−3.7/57	−8.4/86	−7.6/87
Motolimod	Immunology/inflammation	TLR8 agonist	−18.6/61	−4.5/69	−7.0/72	−6.5/70
(−)‐epicatechin gallate	TGF‐β/Smad signaling	Major catechin in green tea	−42.1/99	−6.8/47	−7.8/61	−3.4/49
Etretinate	Retinoid pathway	Retinoid receptor agonist	−41.9/99	−8.1/56	−8.7/67	−3.2/48
PD‐1/PD‐L1 inhibitor 1	Apoptosis	PD‐1/PD‐L1 interaction inhibitor	−41.9/99	−7.4/53	−8.4/65	−3.4/50
Adapalene	Retinoid pathway	RARβ and RARγ agonist	−75.3/90	‐0.5/‐8	−5.5/53	−7.4/66
Nepafenac	Neuroscience	COX‐1 and COX‐2 inhibitor	−78.6/94	−1.9/14	−6.8/60	−6.8/60
Flurbiprofen	Immunology/inflammation	Cyclooxygenase inhibitors	−71.5/67	2.2/‐23	−5.8/55	−6.0/53
Rolipram	Metabolism	PDE4‐inhibitor and an anti‐inflammatory agent	−78.5/95	−1.7/11	−6.4/56	−6.1/54
Ibuprofen	Neuroscience	COX‐1 and COX‐2 inhibitor	−78.3/92	−2.5/17	−6.5/60	−6.7/58
Rapamycin	PI3K/Akt/mTOR signaling	Original antifungal antibiotic	−43.2/71	−2.7/30	−7.9/61	−7.3/73
Everolimus	PI3K/Akt/mTOR signaling	MTOR inhibitor	−46.8/74	−4.2/42	−8.5/69	−6.6/68
Laquinimod	AhR signaling	Immunomodulator	−79.2/97	−1.7/19	−6.2/53	−5.2/53
Clemizole hydrochloride	Neuroscience	H1 histamine receptor antagonist	−74.7/75	−3.1/30	−7.9/71	−7.6/68
Meloxicam	Neuroscience	Nonsteroidal anti‐inflammatory drug	−80.2/98	−1.8/20	−6.1/50	−5.5/51
CO‐1686	JAK/STAT signaling	EGFR inhibitor	−67.4/69	−4.5/49	−9.0/79	−8.3/78
Dexamethasone	Ubiquitination/Proteasome	Glucocorticoidan; anti‐inflammatory	−70.0/90	−2.6/35	−8.3/66	−6.2/64
Mercaptopurine	DNA damage/DNA repair	Purine synthesis inhibitor	−51.3/77	‐0.5/8	−7.6/62	−5.0/52
TAK‐242	Immunology/inflammation	TLR 4 signaling inhibitor	−70.6/57	−1.9/21	−7.6/68	−6.6/59
Betamethasone valerate	Glucocorticoid pathway	Anti‐inflammatory corticosteroid	−65.2/87	−3.6/43	−8.1/65	−5.3/60
Triamcinolone acetonide	Glucocorticoid pathway	Anti‐inflammatory steroid	−66.8/87	−3.0/37	−7.6/59	−7.2/74
ITE	AhR signaling	AHR agonist	−79.1/95	−3.5/40	−7.9/69	−11.5/63
Teriflunomide	Pyrimidine synthesis	Inhibitor of dihydroorotate dehydrogenase	−76.0/93	−2.8/38	−8.7/75	−13.1/79
(S)‐(+)‐Ibuprofen	Prostaglandin synthesis	Inhibitor of Cox‐1 and Cox‐2	−80.3/97	‐0.6/15	−7.5/64	−10.5/61
Etodolac	Neuroscience	COX‐2 inhibitor	−81.3/98	−2.2/30	−6.6/58	−8.6/53
Nizatidine	Neuroscience	Histamine H2 receptor antagonist	−81.1/98	−1.8/27	−6.1/54	−7.8/51
(R)‐(−)‐Ibuprofen	Prostaglandin synthesis	Inhibitor of Cox‐1 and Cox‐2	−81.1/98	−1.5/27	−7.5/66	−10.0/57
Flunixin Meglumin	Neuroscience	Potent cyclooxygenase inhibitor	−81.6/98	−4.6/45	−7.1/62	−9.3/53
Tripelennamine HCl	Neuroscience	H1‐receptor antagonist	−81.3/98	−4.9/48	−7.1/62	−10.2/58
Bepotastine besilate	Neuroscience	Nonsedating, selective antagonist of histamine 1 (H1) receptor	−81.3/98	−2.9/34	−6.2/54	−9.1/53
Antipyrine	Drug metabolism	Analgesic and antipyretic agent	−81.4/99	−5.0/48	−7.9/68	−9.1/55
Salicylic acid	Neuroscience	COX inhibitor	−80.8/98	1.7/4	−5.9/53	−8.8/54
Azathioprine	DNA damage/DNA repair	Purine synthesis and GTP‐binding protein Rac1 activation inhibitor	−56.1/64	−1.1/19	−8.2/70	−11.8/65
GLPG0634	JAK/STAT signaling	JAK1 inhibitor	−77.7/92	−4.8/42	−7.7/66	−13.7/70
Cimetidine	Neuroscience	H2 receptor antagonist	−81.1/98	−2.1/29	−5.8/52	−8.0/52
Deflazacort	GPCR/G protein	Anti‐inflammatory and immunosuppressant	−38.9/90	−5.3/40	−9.7/75	−5.7/84
Acemetacin	Neuroscience	COX inhibitor	−41.8/98	−3.8/25	−7.5/59	−4.5/64
Diclofenac diethylamine	Neuroscience	Nonselective COX inhibitor	−41.5/98	−4.9/30	−7.7/60	−4.3/62
Pranoprofen	Neuroscience	Nonsteroidal COX inhibitor	−41.7/98	−5.3/35	−7.1/55	−4.0/57
Suprofen	Immunology/inflammation	Dual COX‐1/COX‐2 inhibitor	−41.7/98	−7.0/47	−7.7/60	−4.3/63
Vinpocetine	Metabolism	PDE inhibitor	−40.2/95	−7.1/46	−7.7/61	−5.0/70
Alcaftadine	Neuroscience	H1 histamine receptor antagonist	−41.7/98	−6.3/43	−7.2/56	−3.6/52
Hydroxychloroquine sulfate	TLR signaling	Autophagy inhibitor	−41.3/98	−6.6/45	−6.6/50	−3.6/53
MCC950 sodium	Immunology/inflammation	Potent NLRP3 inflammasome inhibitor	−41.8/98	−6.4/44	−8.6/66	−4.0/58
Diclofenac potassium	Immunology/inflammation	Nonsteroidal anti‐inflammatory drug	−41.7/98	−6.9/45	−8.3/65	−4.7/67
Bimatoprost	GPCR/G protein	Prostaglandin analog	−41.7/98	−6.5/44	−7.2/55	−3.6/55
Acetaminophen	Neuroscience	COX inhibitor	−41.4/97	−7.2/48	−7.4/58	−4.2/60
Dimethyl fumarate	Immunology/inflammation	Nuclear factor (erythroid‐derived)‐like 2 (Nrf2) pathway activator	−41.8/98	−7.5/49	−9.0/71	−5.0/70
Dipyridamole	Metabolism	PDE inhibitor	−42.0/98	−7.2/49	−8.7/68	−4.6/65
Sulindac	Neuroscience	Anti‐inflammatory agent;COX inhibitor	−41.5/98	−7.2/48	−7.7/60	−4.6/65
ATB‐346	Neuroscience	Nonsteroidal anti‐inflammatory drug	−41.4/97	−6.6/45	−7.5/58	−3.9/59
Poly(I:C)	Immunology/inflammation	Toll‐like receptor 3 (TLR3) agonist	−41.7/98	−7.0/48	−7.6/59	−3.6/53
Prednisone	Glucocorticoid pathway	Glucocorticoid receptor agonist	−41.7/98	−6.8/47	−7.9/61	−4.3/62
BAF312	GPCR/G protein	S1P agonist,potent and selective	−41.6/98	−6.7/45	−6.6/52	−4.1/60
Indole‐3‐carbinol	AhR signaling	Anticarcinogenic drug	−41.7/98	−7.0/48	−7.0/54	−3.8/54
Mesalamine	Immunology/inflammation	IKK inhibitor	−41.7/98	−5.7/39	−7.3/58	−4.0/56
Pyrantel pamoate	Microbiology and virology	Anthelmintic agent	−41.7/98	−5.6/40	−6.9/53	−3.5/51
(S)‐Naproxen	Neuroscience	Nonselective COX inhibitor	−41.9/99	−4.9/36	−6.9/53	−2.7/42
Methylthiouracil	Thyroid hormone synthesis	Antithyroid preparation	−41.4/97	−3.2/19	−6.6/53	−4.3/60
Camostat mesilate	Membrane Transporter/ion channel	Trypsin‐like protease inhibitor	−40.2/95	−2.2/9	−6.3/51	−4.7/64
Desonide	GPCR/G protein	Nonfluorinated corticosteroid anti‐inflammatory agent	−29.1/75	−3.5/48	−8.2/83	−7.6/89
Asiaticoside	Natural products	Wound healing saponin	−36.6/98	‐0.9/13	−5.3/55	−4.5/55
Ciclesonide	GPCR/G protein	Glucocorticoid	−33.5/90	−2.8/40	−7.2/74	−7.2/85
Loteprednol etabonate	Microbiology and virology	Potent glucocorticoid receptor agonist	−33.4/90	−2.9/41	−7.1/72	−7.1/82
Brucine	Natural products	Highly toxic	−36.5/98	0.5/‐1	−5.2/54	−4.8/58
(+)‐Catechin hydrate	Natural products	Antioxidant flavonoid with various bioactivities	−36.7/98	0.6/‐1	−5.1/52	−5.0/60
Dihydromyricetin	Natural products	Antioxidant and anticancer reagent	−36.5/98	−1.3/24	−5.2/54	−4.7/57
Hesperitin	Natural products	Play role as antioxidant	−36.7/99	‐0.6/14	−5.4/55	−4.7/57
Mycophenolate mofetil	Metabolism	IMPDH inhibitor	−16.1/26	−7.1/64	−8.1/73	−8.5/80
Pimecrolimus	Immunology/inflammation	Inhibitor of inflammatory cytokines secretion, cell‐selective	−10.1/13	−10.3/91	−11.5/99	−17.3/102
Tacrolimus	Microbiology and virology	Macrolide calcineurin inhibitor, immunosuppressant	−5.1/12	−13.9/95	−12.8/100	−6.5/93
Fluocinonide	GPCR/G protein	Glucocorticoid receptor agonist	−16.6/46	−12.2/82	−12.1/95	−6.3/91

*Note:* Each compound was tested at a single concentration (1 µM) in two independent screening runs (*n* = 2), and the median value of both experiments was used for hit identification.

## 4. Discussion

The establishment of a functional multiplex flow cytometry‐based screening assay enables quantitative and functionally diverse profiling of compound‐induced effects on immune cell activation. Current immunological methods often lack the sensitivity, resolution, and throughput necessary to capture the complexity of immune responses, limiting their applicability in early‐phase drug discovery. Compared with highly multiplexed immune profiling technologies such as mass cytometry or cytokine bead array–based approaches, the present assay provides a comparatively accessible and functionally focused platform for simultaneous assessment of immune cell proliferation and activation at the single‐cell level. While high‐dimensional assays offer deeper phenotypic characterization, they are often associated with increased experimental complexity, cost, and data analysis requirements. In contrast, the compact marker panel used here was intentionally designed to balance the biological information content with assay robustness and screening applicability.

By leveraging human PBMCs from both male and female donors, our assay incorporates biologically diverse donor material and provides a functional in vitro model for evaluating immune responses across independent donor samples. Although the present study was not designed to systematically assess sex‐specific differences, the inclusion of donors from both sexes may improve the broader applicability of the screening approach. The inclusion of multiple readouts—T‐cell proliferation (CFSE), activation markers (CD25 and CD71), and B‐cell activation (CD86)—allows for a nuanced evaluation of compound effects across distinct but interrelated facets of immune activation. A deep understanding of these signaling cascades not only helps in deciphering immune responses but also in designing novel immunomodulatory agents that can selectively modulate these pathways for clinical benefit. Importantly, the decision to use anti‐CD3 stimulation without costimulation (e.g., CD28) ensures a dynamic response range sensitive enough to detect both immunostimulatory and immunosuppressive activities without saturating the system, thereby enabling the identification of compounds that either suppress or enhance immune activation.

The determination of the IC_50_ values of immunosuppressive drugs is an essential part of evaluating their potency in modulating immune responses. Validation of our assay using a panel of well‐characterized immunosuppressive drugs confirmed its ability to detect diverse mechanisms of action within T‐cell signaling pathways. The dose–response curves demonstrated distinct potencies and efficacies across the different functional markers for each immunosuppressive drug, underscoring the assay’s resolution in differentiating compound‐specific immunomodulatory profiles and its sensitivity to a range of pharmacological mechanisms. The calculated Z‐factors for CFSE, CD71, and CD25 indicated excellent assay quality, while CD86 showed acceptable performance. The slightly lower Z‐factor for CD86 likely reflects the additional biological variability associated with B‐cell responses in mixed PBMC cultures, where CD86 upregulation depends on T‐cell–mediated activation signals and may therefore vary with donor‐specific immune cell composition.

Conducting a pilot screen with a well‐characterized library enabled the evaluation of assay reproducibility, signal window, and hit discrimination under screening conditions. Importantly, such a library includes compounds targeting diverse immune pathways, providing a robust test of the assay’s capacity to detect a range of modulatory effects on T‐ and B‐cell function. The pilot screen of a 295‐compound library demonstrated the assay’s high sensitivity and specificity. Known immunosuppressants such as cyclosporine A, rapamycin, and dexamethasone were successfully identified as top hits, validating the assay’s pharmacological relevance. The identification of a substantial number of compounds with known mechanisms (e.g., glucocorticoids and COX inhibitors) further supports the assay’s ability to detect both direct and indirect modulators of immune activity. Moreover, the diverse distribution of hits—ranging from compounds active across all markers to those showing specificity for individual readouts—underscores the value of a multiplexed approach in capturing a broad spectrum of immunomodulatory effects. Importantly, the absence of detectable activity for certain compounds with known immunomodulatory properties does not necessarily indicate a lack of biological function but may instead reflect assay‐specific conditions such as stimulation context, exposure time, concentration, donor‐dependent responsiveness, or pathway specificity outside the selected readouts. While many of the top‐ranked compounds in this pilot screen displayed immunosuppressive activity, the assay framework also enables the identification of immune‐activating compounds that enhance T‐cell activation or proliferation beyond the stimulated control. Such compounds may include immune stimulators such as Toll‐like receptor (TLR) agonists or other immunomodulatory agents that enhance antigen‐presenting cell activity or T‐cell signaling pathways, highlighting the potential application of this assay in the discovery of immunostimulatory agents and potential vaccine adjuvants. The combined use of percentage inhibition and robust 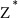
‐scores enabled a dual‐criteria hit selection that integrates biological interpretability with statistical rigor. This strategy minimized the risk of false positives and enhanced reproducibility, as demonstrated by the two‐step duplicate screening approach. Importantly, calculating the 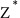
‐score relative to the positive controls—rather than to the global plate median—was critical for correct interpretation. Since strong inhibitors substantially lower the activation level, they may appear as low 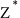
‐scores only when compared to the high baseline of the activated control. In contrast, computing the 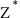
‐score relative to the plate median can underestimate inhibitory effects, especially if many compounds cluster around intermediate activation levels. Thus, the positive control provides a consistent and biologically meaningful reference for identifying both strong inhibitors and compounds that enhance immune activation beyond baseline stimulation.

## 5. Conclusion

In summary, we developed and validated a multiplex flow cytometry‐based screening assay for functional profiling of immunomodulatory compounds in a primary human PBMC model. Validation with clinically relevant drugs and a focused compound library demonstrated its sensitivity, reproducibility, and ability to resolve diverse immunological mechanisms. This platform provides a valuable tool for early‐phase drug discovery and functional immune profiling, supporting the development of targeted and effective immunotherapies. Furthermore, the assay can be combined with cytokine profiling and applied to patient‐derived PBMCs to further explore disease‐specific immune modulation and innate‐adaptive immune interactions, thereby enabling deeper mechanistic insight into the activity of identified compounds.

## Funding

This study was supported by institutional resources provided by the Department of Transfusion Medicine and Hemostaseology, University Hospital Erlangen. No external funding was received, and no funding grant number was applicable. Open Access funding enabled and organized by Projekt DEAL.

## Conflicts of Interest

The authors declare no conflicts of interest.

## Supporting Information

Additional supporting information can be found online in the Supporting Information section.

## Supporting information


**Supporting Information** Table S1. Raw MFI values used for normalization and assessment of assay signal distribution and reproducibility.

## Data Availability

The data that support the findings of this study are available from the corresponding author upon reasonable request.
