# Supplementary material for: Development and Validation of a Multiplex Flow Cytometry‐Based PBMC Assay for Immunomodulator Screening
Source: J Immunol Res. 2026 Aug 4;2026:5535208. doi: 10.1155/jimr/5535208 (PMC13434440; doi:10.1155/jimr/5535208)
Supplement: Supplementary file 1 [file JIMR-2026-5535208-s001.docx]

Supplementary Table 1. Raw MFI values of CFSE, CD25, CD71, and CD86 measured in unstimulated and anti-CD3–stimulated PBMC cultures across three independent experiments. Color coding indicates independent biological replicates: green (experiment 1), blue (experiment 2), and yellow (experiment 3). Data were used for normalization and assessment of assay signal distribution and reproducibility.

| **CFSE** | | **CD25** | | **CD71** | | **CD86** | |
| --- | --- | --- | --- | --- | --- | --- | --- |
| Unstimulated | Stimulated | Unstimulated | Stimulated | Unstimulated | Stimulated | Unstimulated | Stimulated |
| 821700 | 291365 | 382 | 186311 | 881 | 1029899 | 5345 | 30778 |
| 831410 | 300050 | 375 | 214639 | 986 | 1160589 | 4934 | 24765 |
| 831410 | 273134 | 379 | 155758 | 1190 | 1044189 | 4689 | 23030 |
| 843710 | 315412 | 375 | 153630 | 1181 | 1020481 | 4399 | 24841 |
| 853681 | 287962 | 386 | 179996 | 1172 | 1129021 | 3616 | 23239 |
| 871414 | 287118 | 382 | 185670 | 1671 | 1187572 | 3241 | 23592 |
| 868858 | 312645 | 355 | 166283 | 1226 | 997314 | 2957 | 28542 |
| 886906 | 281275 | 361 | 164575 | 2010 | 918210 | 2257 | 25928 |
| 824116 | 290511 | 368 | 170929 | 907 | 869013 | 5172 | 24024 |
| 826541 | 303596 | 361 | 182496 | 925 | 961343 | 4975 | 20509 |
| 826541 | 317270 | 375 | 166283 | 1092 | 1034640 | 4879 | 21888 |
| 836308 | 322911 | 361 | 159007 | 1092 | 992745 | 4556 | 22552 |
| 843710 | 318203 | 344 | 159007 | 1092 | 1048997 | 3968 | 19743 |
| 858710 | 321964 | 355 | 172110 | 2404 | 1025179 | 3509 | 22552 |
| 866310 | 295676 | 358 | 140017 | 1155 | 881058 | 3013 | 24690 |
| 879126 | 291365 | 361 | 143424 | 1905 | 822471 | 2668 | 27156 |
| 781679 | 276361 | 528 | 162884 | 1451 | 873010 | 5839 | 23239 |
| 797916 | 285436 | 483 | 153102 | 1262 | 997314 | 5701 | 21185 |
| 797916 | 315412 | 438 | 142442 | 1524 | 1020481 | 5566 | 24244 |
| 807346 | 306283 | 413 | 168010 | 1199 | 1048997 | 5215 | 22085 |
| 816887 | 290511 | 413 | 168590 | 1235 | 1078315 | 5059 | 21123 |
| 826541 | 291365 | 431 | 148949 | 1369 | 997314 | 4424 | 24540 |
| 836308 | 290511 | 424 | 136693 | 1360 | 922434 | 3724 | 22755 |
| 841235 | 301818 | 434 | 173300 | 1424 | 956940 | 3115 | 30488 |
| 826541 | 279627 | 375 | 136224 | 1119 | 774878 | 4756 | 24318 |
| 814491 | 275551 | 365 | 153102 | 872 | 853211 | 4948 | 21888 |
| 828972 | 299170 | 361 | 160105 | 1262 | 869013 | 4689 | 25612 |
| 831410 | 289659 | 355 | 171518 | 1004 | 893271 | 4689 | 22350 |
| 848681 | 306283 | 355 | 174499 | 1013 | 952556 | 3968 | 24024 |
| 851177 | 286276 | 358 | 196885 | 1119 | 1078315 | 3664 | 28013 |
| 871414 | 269943 | 365 | 177532 | 1460 | 1001905 | 3241 | 25223 |
| 871414 | 305384 | 344 | 197566 | 1137 | 952556 | 2790 | 28991 |
| 627135 | 146110 | 393 | 113994 | 1397 | 501740 | 6220 | 49477 |
| 636413 | 147836 | 368 | 134829 | 628 | 575436 | 6586 | 61370 |
| 642045 | 152243 | 365 | 121237 | 1451 | 547221 | 4838 | 44885 |
| 647727 | 148707 | 358 | 128508 | 1451 | 575436 | 4865 | 52057 |
| 655382 | 148707 | 351 | 147421 | 1551 | 602360 | 3748 | 52770 |
| 653460 | 150023 | 351 | 159555 | 672 | 624812 | 6829 | 57480 |
| 668996 | 147403 | 341 | 140499 | 785 | 580722 | 5258 | 57283 |
| 674916 | 143980 | 358 | 146411 | 2107 | 557316 | 2745 | 65552 |
| 623462 | 156321 | 396 | 124179 | 1727 | 583383 | 5980 | 52235 |
| 623462 | 148271 | 351 | 127193 | 568 | 562433 | 5824 | 42984 |
| 630830 | 153140 | 341 | 133907 | 576 | 591441 | 5566 | 38554 |
| 640162 | 157242 | 337 | 119179 | 637 | 578073 | 5686 | 54229 |
| 651543 | 156781 | 337 | 125033 | 654 | 567598 | 5186 | 51355 |
| 653460 | 152691 | 355 | 131179 | 689 | 575436 | 4810 | 44885 |
| 653460 | 143557 | 341 | 115961 | 689 | 518034 | 4975 | 50492 |
| 667034 | 155405 | 358 | 126324 | 2039 | 506342 | 3357 | 35542 |
| 623462 | 614373 | 386 | 123543 | 715 | 530466 | 6107 | 32533 |
| 638284 | 153590 | 365 | 126324 | 1004 | 602360 | 5447 | 39062 |
| 630830 | 147836 | 372 | 126758 | 646 | 583383 | 6012 | 44587 |
| 634546 | 157242 | 341 | 111298 | 594 | 619121 | 5596 | 48323 |
| 651543 | 150907 | 341 | 115565 | 1388 | 570199 | 4373 | 40234 |
| 649632 | 156781 | 334 | 122909 | 672 | 594152 | 5374 | 52591 |
| 657310 | 144828 | 348 | 113605 | 672 | 532425 | 5855 | 52950 |
| 661182 | 146970 | 334 | 135758 | 724 | 567598 | 4920 | 52770 |
| 630830 | 149145 | 375 | 126758 | 1262 | 562433 | 5701 | 47676 |
| 625295 | 146539 | 358 | 132991 | 550 | 591441 | 5701 | 45036 |
| 636413 | 147403 | 358 | 118366 | 1506 | 549728 | 5229 | 43996 |
| 628979 | 148707 | 361 | 129392 | 568 | 572811 | 6253 | 45186 |
| 645828 | 150023 | 368 | 122489 | 1877 | 547221 | 4743 | 42137 |
| 651543 | 150465 | 368 | 125462 | 689 | 562433 | 5551 | 43559 |
| 657310 | 147836 | 386 | 106100 | 1820 | 572811 | 4193 | 59288 |
| 659243 | 145681 | 389 | 139537 | 2557 | 588742 | 3557 | 56116 |
| 793243 | 237916 | 300 | 305540 | 1693 | 686033 | 6882 | 22961 |
| 786284 | 219777 | 289 | 283103 | 1849 | 998985 | 7207 | 24540 |
| 781679 | 210305 | 286 | 242252 | 2277 | 707525 | 7527 | 19917 |
| 779386 | 217849 | 286 | 297180 | 2082 | 638410 | 7170 | 25223 |
| 781679 | 227662 | 286 | 198935 | 2092 | 978598 | 7281 | 25223 |
| 793243 | 209688 | 286 | 172704 | 2311 | 968562 | 6846 | 19858 |
| 797916 | 213416 | 303 | 167433 | 3158 | 1073763 | 6846 | 21823 |
| 790917 | 221722 | 286 | 282123 | 2277 | 812961 | 6899 | 21888 |
| 797916 | 305384 | 279 | 228418 | 1807 | 591080 | 6368 | 18523 |
| 800263 | 272333 | 293 | 274410 | 1901 | 522608 | 6269 | 20936 |
| 790917 | 243572 | 289 | 248192 | 1964 | 615865 | 6586 | 21502 |
| 788597 | 282932 | 303 | 210236 | 2018 | 714839 | 6846 | 18258 |
| 797916 | 260595 | 279 | 207351 | 2179 | 864775 | 6302 | 19177 |
| 790917 | 225664 | 282 | 264151 | 2136 | 973567 | 6586 | 21565 |
| 795576 | 225664 | 293 | 254279 | 2744 | 919916 | 6451 | 20752 |
| 802617 | 235137 | 286 | 266910 | 1880 | 741043 | 6204 | 22417 |
| 802617 | 316340 | 296 | 231599 | 1765 | 579069 | 5717 | 21374 |
| 809721 | 341443 | 269 | 204506 | 1954 | 905807 | 5581 | 16251 |
| 800263 | 317270 | 289 | 200313 | 2168 | 703896 | 5418 | 16528 |
| 795576 | 293082 | 286 | 226061 | 1943 | 851518 | 5521 | 18576 |
| 804978 | 274743 | 293 | 231599 | 2277 | 978598 | 5949 | 19917 |
| 807346 | 236522 | 286 | 243934 | 2322 | 968562 | 5886 | 22619 |
| 809721 | 225003 | 286 | 269698 | 2212 | 973567 | 5762 | 25534 |
| 814491 | 261361 | 303 | 239751 | 1880 | 873729 | 5345 | 22892 |
| 816887 | 344465 | 310 | 198935 | 1933 | 547292 | 5301 | 20509 |
| 814491 | 280450 | 300 | 230003 | 2028 | 1014555 | 5272 | 19011 |
| 807346 | 243572 | 313 | 222956 | 2501 | 933332 | 6384 | 22755 |
| 809721 | 246451 | 300 | 205924 | 2157 | 1025070 | 5129 | 20389 |
| 807346 | 236522 | 300 | 203099 | 3095 | 1166145 | 5551 | 21185 |
| 812102 | 219777 | 282 | 251652 | 2223 | 884231 | 4770 | 26249 |
| 809721 | 234447 | 296 | 222187 | 2467 | 978598 | 5272 | 20691 |
| 807346 | 260595 | 303 | 237275 | 1922 | 1057279 | 5172 | 24993 |
